# Urinary miR-185-5p is a biomarker of renal tubulointerstitial fibrosis in IgA nephropathy

**DOI:** 10.3389/fimmu.2024.1326026

**Published:** 2024-02-15

**Authors:** Zhi-Yu Duan, Ru Bu, Shuang Liang, Xi-Zhao Chen, Chun Zhang, Qiu-Yue Zhang, Ji-Jun Li, Xiang-Mei Chen, Guang-Yan Cai

**Affiliations:** Department of Nephrology, First Medical Center of Chinese PLA General Hospital, National Key Laboratory of Kidney Diseases, National Clinical Research Center for Kidney Diseases, Beijing Key Laboratory of Kidney Diseases Research, Beijing, China

**Keywords:** IgA nephropathy, miR-185-5p, Urinary biomarkers, renal fibrosis, TJP1

## Abstract

**Background:**

For IgA nephropathy (IgAN), tubular atrophy/interstitial fibrosis is the most important prognostic pathological indicator in the mesangial and endocapillary hypercellularity, segmental sclerosis, interstitial fibrosis/tubular atrophy, and presence of crescents (MEST-C) score. The identification of non-invasive biomarkers for tubular atrophy/interstitial fibrosis would aid clinical monitoring of IgAN progression and improve patient prognosis.

**Methods:**

The study included 188 patients with primary IgAN in separate confirmation and validation cohorts. The associations of miR-92a-3p, miR-425-5p, and miR-185-5p with renal histopathological lesions and prognosis were explored using Spearman correlation analysis and Kaplan-Meier survival curves. Bioinformatics analysis and dual luciferase experiments were used to identify hub genes for miR-185-5p. The fibrotic phenotypes of tubular epithelial cells were evaluated *in vivo* and in HK-2 cells.

**Results:**

miRNA sequencing and cohort validation revealed that the expression levels of miR-92a-3p, miR-425-5p, and miR-185-5p in urine were significantly increased among patients with IgAN; these levels could predict the extent of tubular atrophy/interstitial fibrosis in such patients. The combination of the three biomarkers resulted in an area under the receiver operating characteristic curve of 0.742. The renal prognosis was significantly worse in the miR-185-5p high expression group than in the low expression group (P=0.003). Renal tissue *in situ* hybridization, bioinformatics analysis, and dual luciferase experiments confirmed that miR-185-5p affects prognosis in patients with IgAN mainly by influencing expression of the target gene tight junction protein 1 (TJP1) in renal tubular epithelial cells. *In vitro* experiment revealed that an miR-185-5p mimic could reduce TJP1 expression in HK-2 cells, while increasing the levels of α-smooth muscle actin, fibronectin, collagen I, and collagen III; these changes promoted the transformation of renal tubular epithelial cells to a fibrotic phenotype. An miR-185-5p inhibitor can reverse the fibrotic phenotype in renal tubular epithelial cells. In a unilateral ureteral obstruction model, the inhibition of miR-185-5p expression alleviated tubular atrophy/interstitial fibrosis.

**Conclusion:**

Urinary miR-185-5p, a non-invasive biomarker of tubular atrophy/interstitial fibrosis in IgAN, may promote the transformation of renal tubular epithelial cells to a fibrotic phenotype via TJP1.

## 1 Introduction

IgA nephropathy (IgAN) is one of the most common forms of primary glomerulonephritis worldwide. Patients with IgAN generally have a poor prognosis. Recent data from the United Kingdom National Registry of Rare Kidney Diseases (RaDaR) IgAN cohort study showed that most patients with IgAN progress to end-stage kidney disease within 10-15 years; nearly all patients have a risk of eventual progression to end-stage kidney disease within their expected lifespan (1). Tubular atrophy and interstitial fibrosis are among the most important pathological types of IgAN (2). Across multiple IgAN prediction models, including the International IgAN Prediction Tool (IIgANPT), tubular atrophy/interstitial fibrosis is the most important prognostic pathological indicator in the mesangial and endocapillary hypercellularity, segmental sclerosis, interstitial fibrosis/tubular atrophy, and presence of crescents (MEST-C) score (3, 4). Tubular atrophy and interstitial fibrosis are often long-term chronic processes. In the early stages of the disease, the renal tubulointerstitium is almost normal; in advanced stages of disease, tubular injury causes a fibroproliferative peritubular response, mononuclear inflammatory cell infiltration, and eventual establishment of interstitial fibrosis and tubular atrophy (5). Renal biopsy pathology at a single time point cannot determine the statuses of renal tubulointerstitial lesions throughout the course of disease. The identification of non-invasive biomarkers for tubular atrophy and/or interstitial fibrosis can help nephrologists to monitor renal tubulointerstitial disease progression in their patients at any time.

MicroRNAs (miRNAs) are a group of non-coding single-stranded small molecule RNAs that can regulate gene expression through post-transcriptional degradation of messenger RNAs (mRNAs) or translational repression of protein synthesis (6). Some studies have shown that miRNAs, such as miR-21 (7, 8), miR-150 (9), and miR-192 (10), can be used to predict interstitial fibrosis and tubular atrophy. In addition, anti-fbrotic miRNAs, such as miR-21 and miR-let-7 families, also play a major role in renal fibrosis (11). MiR-let-7 family members were involved in the antifibrotic mechanism of AcSDKP (12) whereas, miR-29 family members were implicated in the antifibrotic mechanism of linagliptin (DPP-4 inhibitor) (13). AcSDKP may work by inhibiting higher levels of DPP-4 and restore anti-fibrotic miRNAs crosstalk between miR-29s and miR-let-7s (14). However, these miRNA biomarkers are derived from renal tissue, plasma, or serum. Urinary miRNAs, which pass through or directly originate from the kidneys and are easy to collect non-invasively, represent a promising source of non-invasive IgAN biomarkers. Urinary miR-25-3p, miR-144-3p, miR-486-5p, and miR-16-5p have been identified as diagnostic biomarkers for IgAN (15, 16). Urinary miR-25-3p, miR-144-3p, and miR-486-5p are mainly derived from urinary red blood cells (16). Urinary miR-16-5p can be used to predict endocapillary hypercellularity in IgAN (15). In 2012, one study showed that urinary miR-21, miR-29b, miR-29c, and miR-93 are involved in the transforming growth factor (TGF)- β1/mothers against decapentaplegic homolog 3 (SMAD3) pathway, and that miR-93 is associated with pathological glomerular scarring; these miRNAs may contribute to renal fibrosis within IgAN (17). Our subsequent exploratory research revealed that urinary miR-205 and miR-21 can be used to distinguish patients with IgAN displaying severe tubular atrophy/interstitial fibrosis from patients displaying mild tubular atrophy/interstitial fibrosis (18). However, the above studies had sample sizes of <60 patients; to our knowledge, there has been limited research regarding the mechanisms of miRNA contribution to tubular atrophy/interstitial fibrosis. The present study included 188 patients with IgAN, and utilized a median follow-up interval of 5 years. The mechanisms of miRNAs effects were identified in animal models and cell experiments, providing a basis for future clinical applications of urinary miRNAs in predicting tubular atrophy/interstitial fibrosis among patients with IgAN.

## 2 Methods

### 2.1 Study design and sample inclusion

This single center observational study included 188 individuals diagnosed with primary IgAN at the First Medical Center of the People’s Liberation Army General Hospital during the period from January 2013 to October 2014. The inclusion criteria were age>16 years, confirmation by renal biopsy, and estimated glomerular filtration rate (eGFR)>15 mL/min/1.73 m^2^ before renal biopsy. Patients were excluded if they had secondary IgAN (e.g., Henoch-Schoenlein purpura nephritis or lupus nephritis), urinary tract infection or pregnancy before renal biopsy. There were no interventions or restrictions concerning clinical treatment. Clinical and demographic characteristics of patients in IgAN and control groups are shown in **Supplementary Table 2**.

Thirty-three age- and sex-matched healthy individuals served as the normal control group. The study protocol was approved by the ethics committee of the Chinese People’s Liberation Army General Hospital (Beijing, China) (approval no. S2014–004-002) in accordance with the Declaration of Helsinki. Informed consent was obtained from each patient before inclusion in the study.

### 2.2 Urine collection and miRNA extraction

On the morning before renal biopsy, 50-100ml of urine were collected from each patient and either immediately discarded or stored at -4°C within 4 h. Each urine sample was centrifuged at 3000 g for 30 min and 13000 g for 15 min at 4°C. Urinary sediment was stored at -80°C until use. TRIzol (Invitrogen) was used to extract total RNA from urinary sediment, in accordance with the manufacturer’s protocol. RNA concentration and purity were determined using a NanoDrop 2000c spectrophotometer (Thermo Fisher Scientific, Waltham, MA, USA).

### 2.3 miRNA microarray and reverse transcription polymerase chain reaction (RT-PCR) analysis

In our previous studies (16, 19), miRNA microarray data were acquired from nine patients with IgAN and three healthy participants using Agilent Human miRNA Microarrays (V19.0). Moreover, our previous miRNA expression profile results were used for screening (GSE64306) (19).

RT-qPCR analysis of urinary sediment was conducted using an miRcute miRNA First-Strand cDNA Synthesis Kit (Tiangen Biotech, Beijing, China) and an miRcute miRNA qPCR Detection kit (Tiangen Biotech); the analysis focused on hsa-U6, hsa-miR-92a-3p, hsa-miR-425-5p, and hsa-miR-185-5p. All urinary miRNA primers were purchased from Tiangen Biotech. For HK-2 cells and the unilateral ureteral obstruction (UUO) model, reverse transcription was conducted with SuperScript III Reverse Transcriptase (ABI-invitrogen), in accordance with the manufacturer’s protocol. RT-qPCR analysis was performed with SYBR qPCR mix (ABI-Invitrogen). All RT-qPCR protocols utilized the ABI Prism^®^ 7900 Sequence Detection System (Applied Biosystems, Foster City, CA, USA). Based on our previous research, we used U6 (20) and glyceraldehyde-3-phosphate dehydrogenase (GAPDH) as housekeeping genes. Relative miRNA expression levels were calculated using the ΔΔCt method.

### 2.4 Clinical characteristics and renal histopathological assessment

Patient demographic and clinical characteristics were recorded at the time of renal biopsy, including age, sex, mean arterial pressure, urine osmolality, blood albumin, baseline serum creatinine, blood uric acid, baseline eGFR, hematuria, and proteinuria. Hematuria was defined as >5 red blood cells/high-power field (assessed manually) or >28 red blood cells/μL (assessed by automated device). Renal histopathological lesion severity in patients with IgAN was evaluated by two experienced pathologists using the Oxford classification system (21): mesangial hypercellularity (M0, ≤0.5; M1, >0.5), endocapillary hypercellularity (E0, absent; E1, present), segmental glomerulosclerosis (S0, absent; S1, present), and tubular atrophy/interstitial fibrosis (T0, ≤25%; T1, 25–50%; T2, ≥50%). Renal function and urinary protein remission were also monitored (22). GFR was estimated by the Chronic Kidney Disease Epidemiology Collaboration (CKD-EPI) equation (23). The renal endpoint was defined as a 50% decrease in eGFR from baseline (the time of renal biopsy) or progression to end-stage renal disease (i.e., eGFR <15 mL/min/1.73 m^2^, initiation of renal replacement therapy, or kidney transplantation).

### 2.5 Fluorescence *in situ* hybridization

Cy3-labeled probe sequences of miR-185-5p was prepared by GENERAL BIOL Co. (Chuzhou, China). Renal biopsy tissues (length: 0.3–0.5 cm) were collected from patients with IgAN, then used to prepare paraffin sections. For antigen recovery, paraffin sections were placed in 1× citric acid buffer (pH 6.0) and heated in a microwave on high power for 6 min, switched to medium power for 10 min, and cooled to room temperature. Next, 3% H_2_O_2_ was added to the sections in a dropwise manner, and they were incubated at room temperature in the dark for 15 min to block endogenous peroxidases. Pre-hybridization solution (1:100 dilution of salmon semen) was then added in a dropwise manner, and the sections were incubated at 37°C for 1 h. Subsequently, sections were incubated with 2 μM hsa-mir-185-5p probe hybridization solution (added in a dropwise manner) overnight at 42 °C. Sections were then washed with 4×citric acid buffer, 2×citric acid buffer, and 1×citric acid buffer at 37°C for 10 min respectively; these washes removed unbound hsa-mir-185-5 probe.

### 2.6 Cell culture and transfection

HK-2 cells were obtained from Pricella (Wuhan, China) and cultured in Dulbecco’s modified Eagle medium supplemented with 10% fetal bovine serum, 100 U/mL penicillin, and 0.1 mg/mL streptomycin. Cells were plated in six-well plates and cultured until they reached 60%-70% confluence, then cultured in serum-free medium for an additional 12 h before transfection. HK-2 cells were transfected with 80 nM control mimic or miR-185-5p mimic (GenePharma, China) using Lipofectamine 3000 (Invitrogen), in accordance with the manufacturer’s protocol.

HK-2 cells were cultured in serum-free conditions overnight, then incubated with 8 ng/mL recombinant human TGF-β1 for 48 h to induce epithelial-mesenchymal transition. TGF-β1 stimulated HK-2 cells were treated with 80 nM control inhibitor or miR-185-5p inhibitor using Lipofectamine 3000 for an additional 48 h.

The hsa-miR-185-5p mimic and hsa-miR-185-5p inhibitor sequences were as follows: hsa-miR-185-5p mimic, UGGAGAGAAAGGCAGUUCCUGA; hsa-miR-185-5p inhibitor, UCAGGAACUGCCUUUCUCUCCA.

### 2.7 Establishment of UUO mouse model

C57BL/6N male mice (6-8 weeks, 18-25 g) were housed under a constant temperature (18-22 °C), constant humidity (50 ± 5%), and 12 h light/dark cycle. They were randomly divided into four groups: sham operation, UUO, UUO+miR-185-5p agomir group, and UUO+miR-185-5p antagomir (n=6 per group). Mice in the UUO groups were anesthetized by intraperitoneal injection of pentobarbital (50 mg/kg); the abdominal wall skin, muscles, and peritoneal tissue were incised in the middle of the abdomen. A blunt forceps was used to separate tissue surrounding the ureter and expose the left ureter. The ureter was ligated with 4-0 threads; the peritoneum, muscular layer, and skin layer were sutured in a layer-by-layer manner. On days 10 and 13 after ureteral ligation, 40 mg/kg mmu-miR-185-5p agomir or mmu-miR-185-5p antagomir was administered by intravenous injection. On day 14 after surgery, mice were euthanized and tissue was collected from the left kidney. In the sham operation group, the left ureter was exposed without ligation; other methods were identical to procedures in the UUO group.

The sequences of mmu-miR-185-5p agomir (the modification of agomir in the antisense chain, cholesterol modification is performed at the 3 ‘end, two thioskeleton modifications are performed at the 5’ end, four thioskeleton modifications are performed at the 3 ‘end, and full chain methoxy modification is performed) and mmu-185-5p antagomir (cholesterol modification is applied to the 3 ‘end of antogimir, with two thioskeleton modifications at the 5’ end, four thioskeleton modifications at the 3 ‘end, and full chain methoxy modification) sequences were as follows: mmu-miR-185-5p agomir, sense, UGGAGAGAAAGGCAGUUCCUGA; mmu-miR-185-5p agomir, anti-sense, AGGAACUGCCUUUCUCUCCAUU. mmu-185-5p antagomir, UCAGGAACUGCCUUUCUCUCCA.

### 2.8 Masson’s trichrome staining and Sirius red staining

Renal tissue was fixed in 10% neutral formaldehyde, dehydrated with ethanol, embedded in paraffin, and cut into 4 μm sections. Sections were stained with hematoxylin solution, followed by Biebrich scarlet acid fuchsin solution. Next, the sections were counterstained in phosphotungstic acid solution. After removal of phosphomolybdate, sections were directly transferred to aniline blue for 5-10 min, then cleared in 1% acetic acid solution for 2–5 min. Finally, sections were dehydrated with 95% alcohol and anhydrous alcohol, cleared in xylene, mounted with resin mounting agent and observed under an optical microscope.

Paraffin-embedded renal tissue sections were deparaffinized hydrated, and washed. Next, they were soaked in 1% Sirius red/saturated picric acid solution for 1 h, rinsed with 0.5% acetic acid, dehydrated, and sealed with mounting agent. Finally, they were observed under an optical microscope.

### 2.9 Luciferase reporter assay

293T cells (10^5^) were transfected with 3’-untranslated region (UTR) luciferase reporter constructs (3′UTR-NC, 3′UTR-TJP1, or 3′UTR-TJP1-mutant), miRNA (miRNA-NC, miR-185-5p-mimic, or miR-185-5p-inhibitor), and Renilla luciferase using Lipofectamine 2000 (Invitrogen). After transfection for 48 h, luciferase activities were measured using a Dual Luciferase Assay Kit (Beyotime Biotechnology) and microplate reader (SpectraMax M5). These experiments were performed three times.

### 2.10 Western blotting

HK-2 cells and renal tissues were homogenized in radioimmunoprecipitation assay lysis buffer (Thermo Fisher Scientific) and protein concentrations were detected using a bicinchoninic acid assay (Thermo Fisher Scientific). Proteins were separated by 10% sodium dodecyl sulfate–polyacrylamide gel electrophoresis (Thermo Scientific) and transferred to polyvinylidene fluoride membranes (Millipore). The membranes were blocked with bovine serum albumin for 1 h, then incubated with the following primary antibodies overnight: anti-TJP1 (8193, Cell Signaling), anti-fibronectin (26836, Cell Signaling), anti-α-smooth muscle actin (α-SMA; 19245, Cell Signaling), anti-collagen I (COL-I; ab138492, Abcam), anti-collagen III (COL-III; ab184993, Abcam), and anti-GAPDH (bsm-0978M, Bioss). Goat anti-rabbit secondary antibody (Cell Signaling Technology) was used for detection on a ChemiScope6100 (Clinx Science Instruments). The relative protein expression levels of the above indicators were normalized to GAPDH.

### 2.11 Statistical analysis

Statistical analysis and graphical representation were performed using SPSS 24.0 (IBM Corp., Armonk, NY, USA) and GraphPad Prism 5.01 for Windows (GraphPad Software Inc., San Diego, CA, USA). Continuous variables were expressed as means ± standard deviations; they were compared using independent samples t-tests. Non-normally distributed data were expressed as medians and interquartile ranges; they were compared by the nonparametric Mann–Whitney U test. Differences in urinary miRNAs were compared among the IgAN, disease control, and normal control groups by one-way analysis of variance or the Kruskal–Wallis test. Categorical variables were expressed as frequencies (percentages) and compared using the chi-squared test or Fisher’s exact test, as appropriate. The Shapiro–Wilk test was utilized to assess the normality of continuous data. Pearson or Spearman correlation coefficients were used to analyze correlations. Receiver operating characteristic (ROC) curves were constructed and areas under the ROC curve (AUCs) were calculated to investigate the utility of each urinary miRNA in predicting IgAN and evaluating histological injury. Kaplan–Meier curves were used to analyze renal survival in conjunction with the log-rank test. P-values <0.05 were considered statistically significant. All tests of probability were two-tailed.

## 3 Results

### 3.1 miRNA profiling by microarray analysis

**Figure 1:**
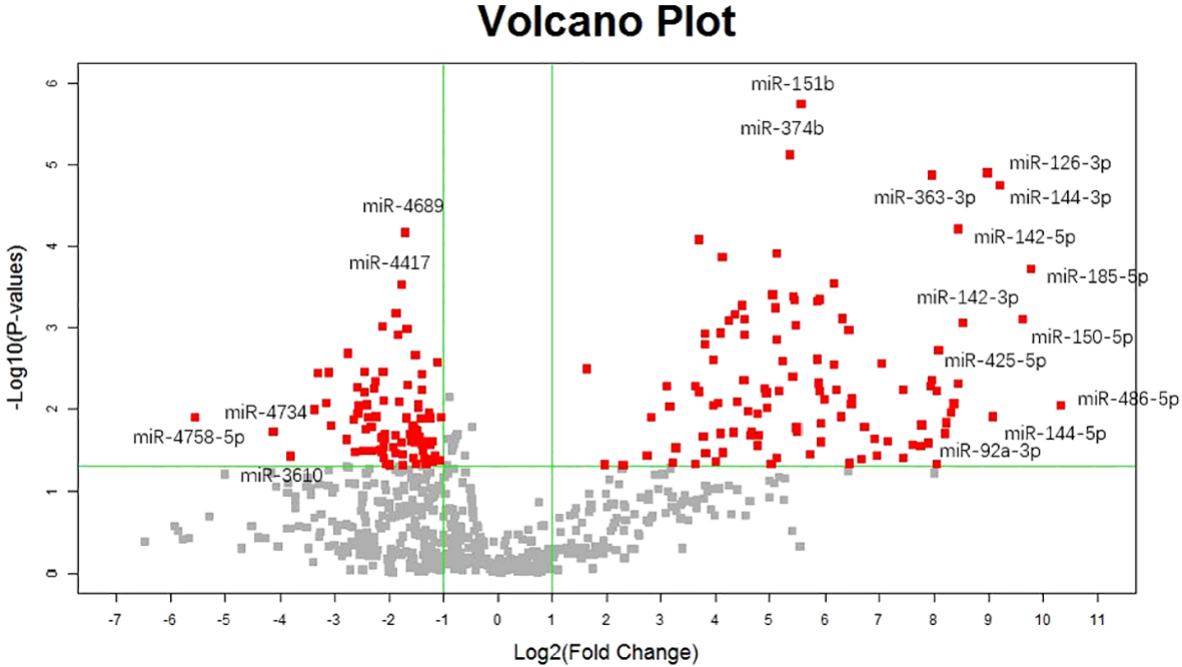
Volcano plot of diffrentially urinary miRNAs between IgA nephropathy and healthy control.

Our previous studies revealed two urinary miRNA profiles in patients with IgAN (16, 19). Three urinary miRNAs with consistent patterns (miR-92a-3p, miR-425-5p, and miR-185-5p) were included in a combined analysis of these expression profiles (**Supplementary Table 1**). Across both profiles, the expression of these three urinary miRNAs was significantly higher in the IgAN group than that in the normal control group (**Figure 1**).

### 3.2 Comparison of urinary miRNAs between IgAN and control groups

To confirm the results of expression profiling, we established a confirmation cohort consisting of 40 patients with IgAN and 33 healthy normal control individuals, matched by age and sex. The levels of urinary miR-92a-3p, miR-425-5p, and miR-185-5p were significantly higher in the IgAN group than in the healthy normal control group (P<0.001); these trends were consistent with the chip results (**Table 1**).

**Table 1:** Comparison of urinary miRNA levels in the confirmation cohort.

Group	miR-92a-3p	miR-425-5p	miR-185-5p
IgAN (n=40)	0.1302 (0.0591-0.3931)	0.0203 (0.0094-0.0469)	0.0184 (0.0073-0.0577)
NC (n=33)	0.0399 (0.0205-0.0615)	0.0077 (0.0042-0.0094)	0.0026 (0.0013-0.0044)
P value	<0.001	<0.001	<0.001

NC, normal control group. Relative microRNA expression levels were expressed as 2^−ΔΔCT^.

### 3.3 Relationships of urinary miRNAs with tubular atrophy/interstitial fibrosis

The expression levels of urinary miR-92a-3p, miR-425-5p, and miR-185-5p were significantly higher in patients with IgAN who had renal tubulointerstitial lesions (T1-T2) than in patients who lacked renal tubulointerstitial lesions (T0) in a confirmation cohort (**Table 2**).

**Table 2:** Comparison of urinary miRNA levels in different renal tubulointerstitial lesions among patients with IgAN.

	confirmation cohort		P value	validation cohort		P value
	T1-T2 (n=24)	T0 (n=16)		T1-T2 (n=83)	T0 (n=65)	
miR-92a-3p	0.2262 (0.1067-0.5019)	0.0707 (0.0282-0.1790)	0.002	0.1740 (0.0811-0.3738)	0.0957 (0.0524-0.3691)	0.001
miR-425-5p	0.0312 (0.0137-0.1222)	0.0105 (0.0069-0.0296)	0.003	0.0207 (0.0122-0.0451)	0.0133 (0.0085-0.0233)	0.003
miR-185-5p	0.0293 (0.0173-0.0999)	0.0081 (0.0037-0.0179)	<0.001	0.0282 (0.0104-0.0896)	0.0120 (0.0066-0.0230)	<0.001

Relative microRNA expression levels were expressed as 2^−ΔΔCT^.

Moreover, to validate the above results, we established a validation cohort consisting of 148 patients with IgAN (83 with T1-T2 grade, 65 with T0 grade). Similar to the previous assay, the levels of urinary miR-92a-3p, miR-425-5p, and miR-185-5p were significantly higher in the T1-T2 group than in the T0 group (**Table 2**).

Furthermore, the AUCs of urinary miR-92a-3p, miR-425-5p, and miR-185-5p for predicting renal tubulointerstitial lesions (T1-T2) in patients with IgAN were 0.684, 0.679, and 0.739, respectively (**Table 3**). When the three indicators were combined, the AUC was 0.742, sensitivity was 63.8%, and specificity was 76.5%.

**Table 3:** ROC curves for urinary miRNAs in predicting renal tubulointerstitial lesions among patients with IgAN.

	AUC	95% CI	P Value	Optimal cut-off value	Sensitivity (%)	Specificity (%)
miR-92a-3p	0.684	0.608-0.761	<0.001	0.1167	68.6	69.1
miR-425-5p	0.679	0.602-0.755	<0.001	0.0133	76.2	54.3
miR-185-5p	0.739	0.668-0.809	<0.001	0.0256	55.2	85.2

Univariate logistic regression analysis showed that MAP, proteinuria, urine osmolality, blood albumin, baseline serum creatinine, blood uric acid, cystatin C, and baseline eGFR were predictive indicators of tubular atrophy/interstitial fibrosis in IgA nephropathy. When the above clinical indicators were combined with three miRNAs biomarkers, the AUC was 0.881, the sensitivity was 76.1%, and the specificity could be improved to 96.1% (P<0.001).

### 3.4 Urinary miRNAs in the prognosis of IgAN

**Figure 2:**
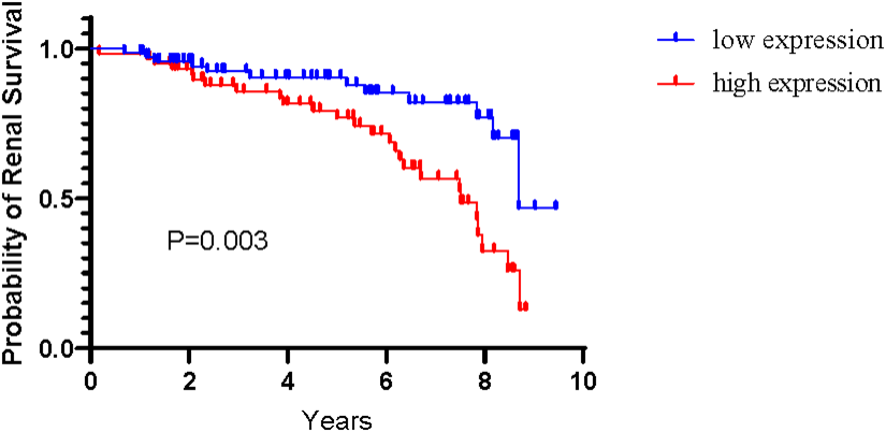
Renal survival among patients with IgAN according to level of urinary miR-185-5p.

Among 188 patients with IgAN, 134 patients completed more than 1 year of follow-up, two patients progressed to end-stage renal disease within <1 year. During mean follow-up interval of 5 years (inter-quartile range, 2.26-7.45 years), 38 patients reached the renal endpoint. The expression level of urinary miR-185-5p was significantly higher among patients in the progressive group (i.e., patients who reached the renal endpoint) than among patients in the non-progressive group [0.0292 (0.0149-0.0886) vs 0.0123 (0.0069-0.0247), P<0.0001]. According to the median expression level of urinary miR-185-5p, patients with IgAN were divided into a high expression group and a low expression group. The renal prognosis was significantly worse in the high expression group than in the low expression group (P=0.003, **Figure 2**).

### 3.5 TJP1 is a direct target gene for miR-185-5p

**Figure 3:**
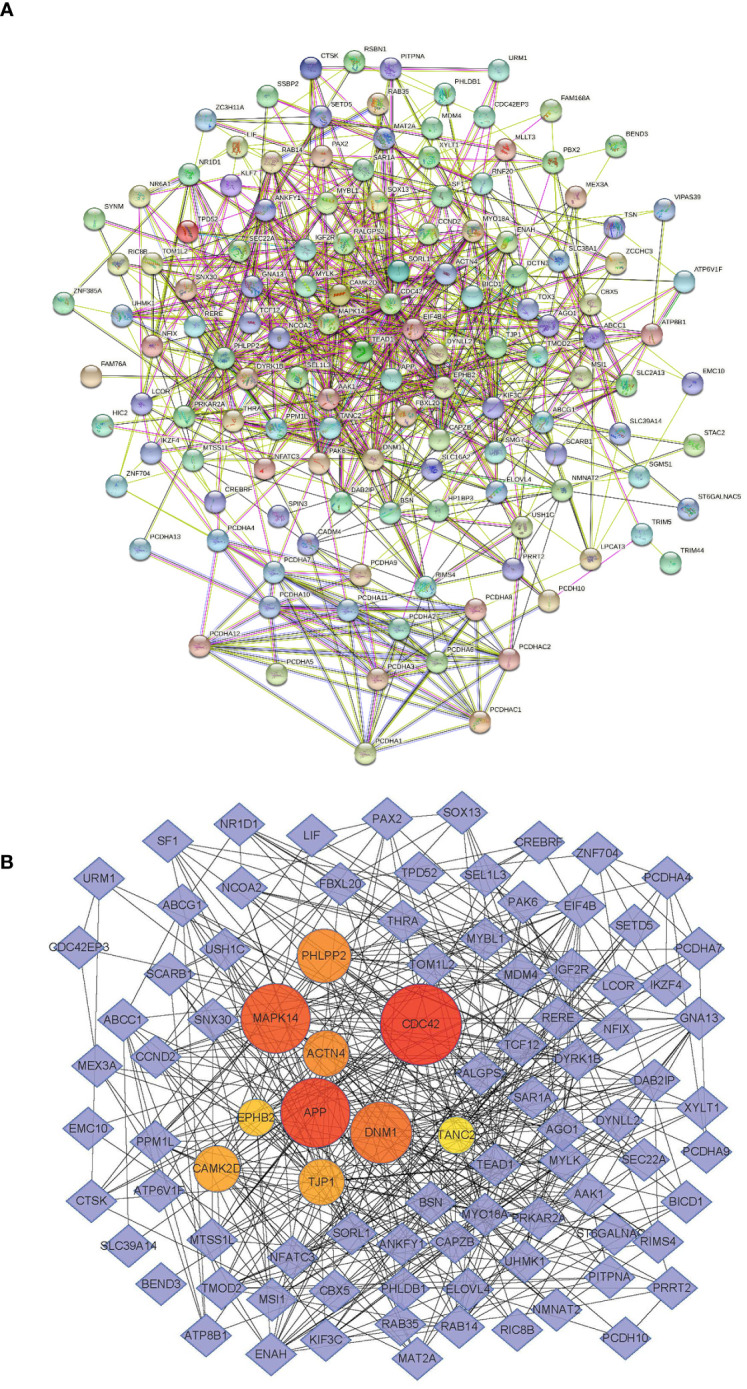
miR-185-5p target genes. **(A)** PPI network of miR-185-5p target genes. **(B)** miR-185-5p hub genes.

We searched for miR-185-5p target genes in three databases: starBase (24), miRDB (25), and TargetScan (26). Our search revealed 138 predicted target genes (**Supplementary Figure 1**). Gene Ontology (GO) analysis revealed the top 10 terms in these three fields (**Supplementary Figure 2**). Kyoto Encyclopedia of Genes and Genomes (KEGG) analysis also identified pathways related to the functions of common target genes (**Supplementary Figure 3**). There were 18 pathways associated with the functions of 138 target genes. Among these pathways, actin cytoskeleton regulation, focal adhesion, and adherens junctions all play important roles in kidney pathophysiology, including epithelial–mesenchymal transition. Additionally, we used the String database (https://stringdb.org) to establish protein–protein interaction networks (**Figure 3A**) for the 138 target genes; we used Cytoscape software (https://cytoscape.org) to identify hub genes (**Figure 3B**) among the target genes. These analyses showed that the epithelial marker TJP1 was an important hub gene.

We also confirmed the targeting relationship between miR-185-5p and TJP1 using a dual luciferase reporter gene assay (**Supplementary Figure 4**). Compared with co-transfection of miR-185-5p and PmirGLO-TJP1-mutant type (TJP1-MUT), the co-transfection of miR-185-5p and PmirGLO-TJP1-wild type (TJP1-WT) led to significantly lower fluorescence intensity. Furthermore, the TJP1 expression level was substantially increased after transfection with miR-185-5p inhibitor (**Supplementary Figure 4**). These results clearly demonstrated that TJP1 is a downstream target of miR-185-5p.

### 3.6 Groupwise comparison of TJP1 expression levels in renal tissue

Considering the previous research findings, we used mass spectrometry to compare TJP1 expression levels in renal tissue between the IgAN group (n=59) and the normal control group (n=19). We found that the TJP1 expression level was significantly lower in the IgAN group (0.919 ± 0.156) than in the normal control group (1.053 ± 0.193, P=0.003) (27). The expression level of COL-I in renal tissue was significantly higher in the IgAN group [1.612 (0.753-2.474)] than that in the normal control group [1.007 (0.909-1.386), P=0.039]. Similarly, the expression level of TGF-β1 in renal tissue was significantly higher in the IgAN group [1.128 (0.961-1.893)] than in the normal control group [0.796 (0.756-1.082), P=0.006].

### 3.7 Distribution of TJP1 in renal tissue

According to the Human Protein Atlas (https://www.proteinatlas.org), TJP1 is mainly expressed in renal tubules. *In situ* hybridization staining revealed that miR-185-5p is also localized in renal tubular epithelial cells (**Supplementary Figure 5**).

### 3.8 miR-185-5p promotes tubulointerstitial fibrosis via TJP1 *in vitro*

**Figure 4:**
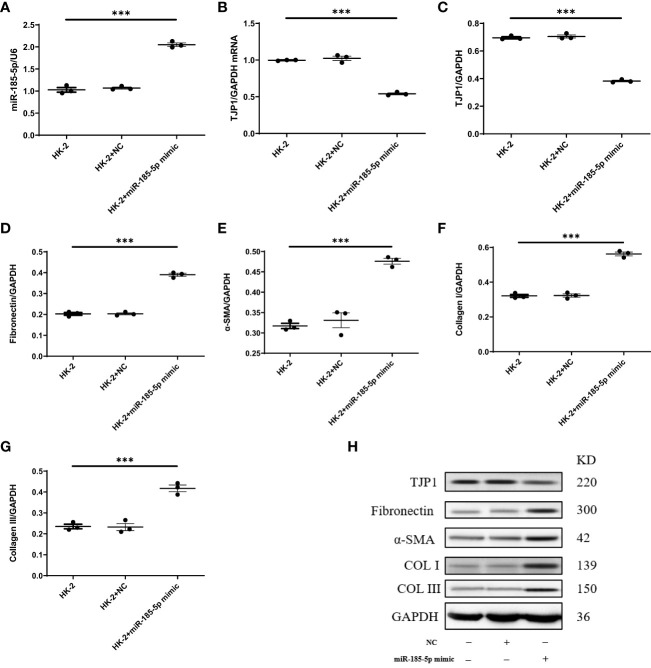
Upregulation of miR-185-5p can promote the expression of fibrotic phenotype-related proteins in HK-2 cells. **(A)** miR-185-5p expression in HK-2 by RT-PCR. **(B)** mRNA expression of TJP1 in HK-2 by RT-PCR. **(C)** Quantification of TJP1 by western blotting in HK-2. **(D)** Quantification of Fibronectin by western blotting in HK-2. **(E)** Quantification of α-SMA by western blotting in HK-2. **(F)** Quantification of Collagen I by western blotting in HK-2. **(G)** Quantification of Collagen III by western blotting in HK-2. **(H)** Representative western blotting of these protein (TJP1, Fibronectin, α-SMA, Collagen I, and Collagen III). ***P (vs. normal control HK-2 group)<0.001.

**Figure 5:**
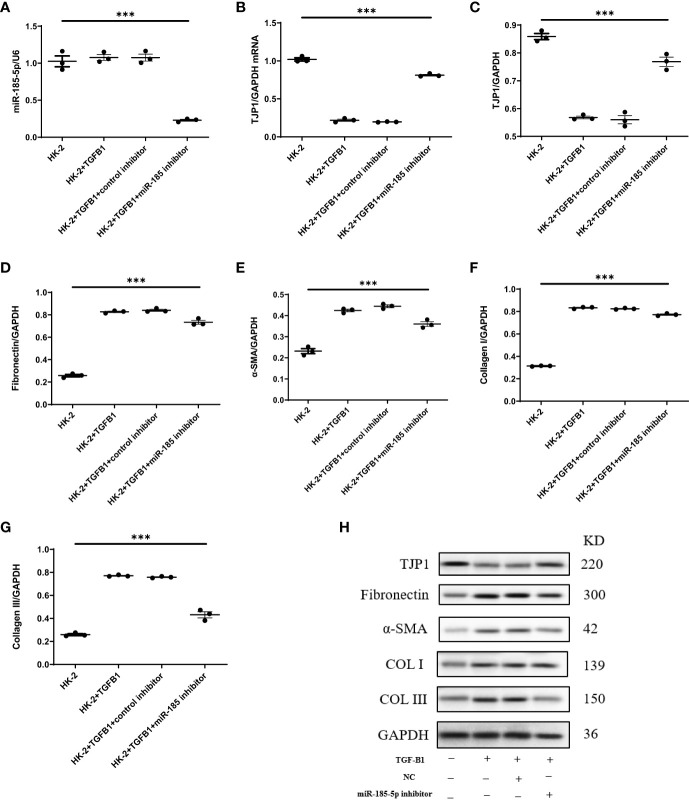
Downregulation of miR-185-5p can inhibit the expression of fibrotic phenotype-related proteins in HK-2 cells. **(A)** miR-185-5p expression in HK-2 by RT-PCR. **(B)** mRNA expression of TJP1 in HK-2 by RT-PCR. **(C)** Quantification of TJP1 by western blotting in HK-2. **(D)** Quantification of Fibronectin by western blotting in HK-2. **(E)** Quantification of α-SMA by western blotting in HK-2. **(F)** Quantification of Collagen I by western blotting in HK-2. **(G)** Quantification of Collagen III by western blotting in HK-2. **(H)** Representative western blotting of these protein (TJP1, Fibronectin, α-SMA, Collagen I, and Collagen III). ***P (vs. normal control HK-2 group)<0.001.

To explore the mechanism by which miR-185-5p contributes to renal tubulointerstitial lesions in IgAN, we transfected HK-2 cells with miR-185-5p mimic or control mimic. RT-qPCR revealed a significant increase in miR-185-5p expression in the miR-185-5p mimic group (P<0.001, **Figure 4A**), indicating successful transfection. The TJP1 expression level was significantly lower in the miR-185-5p mimic group than in the blank group or the control mimic group (P<0.001, **Figure 4B**). Western blotting demonstrated that miR-185-5p overexpression could inhibit TJP1expression (**Figure 4C**), while promoting the expression of α-SMA, fibronectin, COL-I, and COL-III (**Figures 4D–G**). Moreover, stimulation of HK-2 cells with TGF-β1 for 48 h led to increased expression of α- SMA, fibronectin, COL-I, and COL-III (**Figures 5D–G**), along with decreased expression of TJP1 (P<0.001, **Figures 5B, C**). Transfection with miR-185-5p inhibitor could partially restore TJP1 expression while downregulating the expression levels of α-SMA, fibronectin, COL-I, and COL-III (**Figures 5D–G**). These results indicated that miR-185-5p could promote fibrotic phenotype in renal tubular epithelial cells through TJP1 expression *in vitro*.

### 3.9 miR-185-5p exacerbates tubulointerstitial fibrosis in UUO model mice

**Figure 6:**
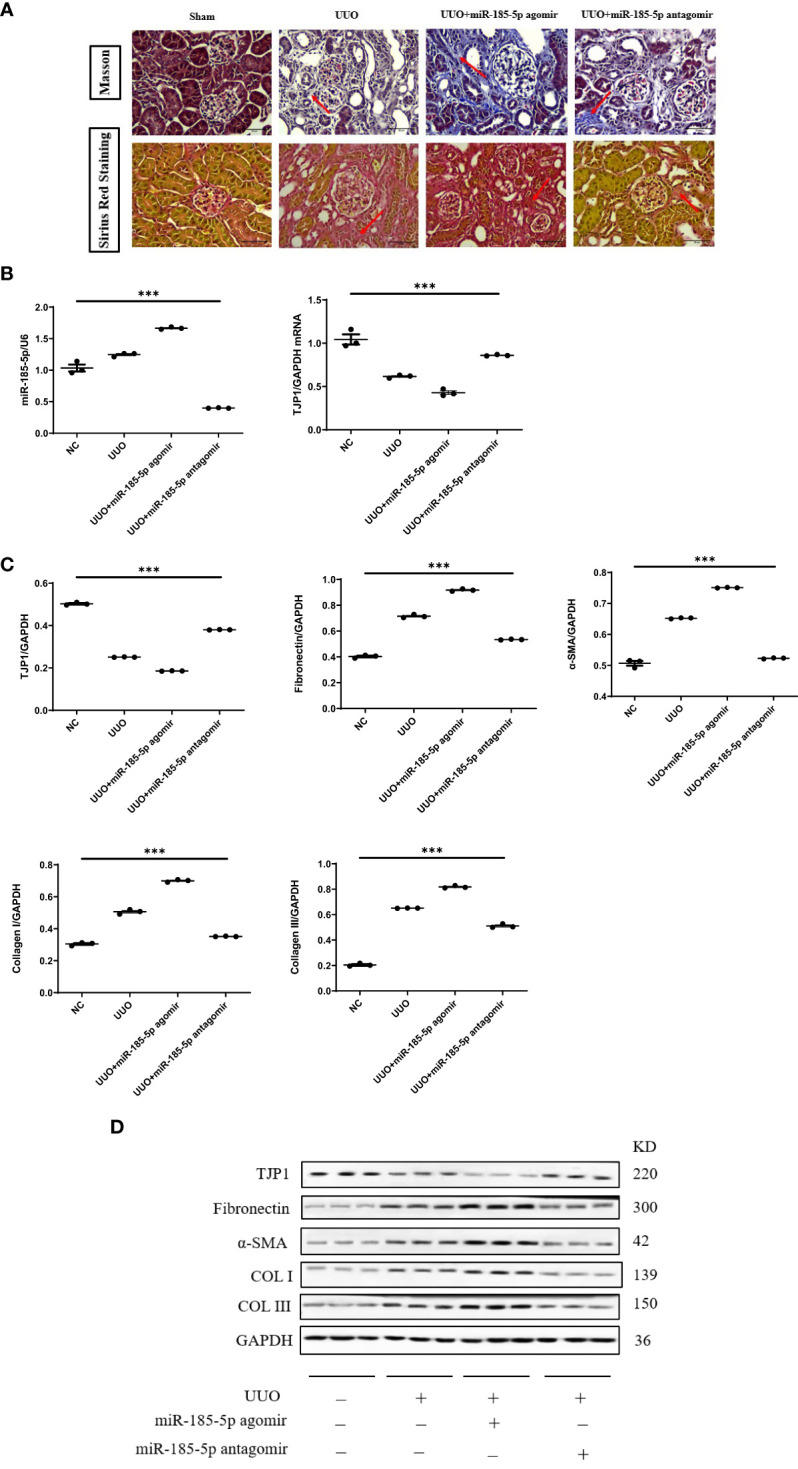
miR-185-5p promotes tubulointerstitial fibrosis through TJP1 in UUO model mice. **(A)** Masson’s trichrome and Sirius red staining of renal tissues as observed under a light microscope (× 400). **(B)** miR-185-5p and TJP1 mRNA expression in renal tissues by RT-PCR. **(C)** Quantification of TJP1, Fibronectin, α-SMA, Collagen I and Collagen III by western blotting in renal tissues. **(D)** Representative western blotting of these protein (TJP1, Fibronectin, α-SMA, Collagen I, and Collagen III). ***P (vs. normal control HK-2 group)<0.001.

Next, we investigated the functional role of miR-185-5p in UUO model mice. Both Masson’s trichrome staining and Sirius red staining showed that transfection with miR-185-5p agomir exacerbated tubulointerstitial fibrosis, whereas transfection with miR-185-5p antagomir alleviated tubulointerstitial fibrosis in UUO model mice (**Figure 6A**). Compared with the sham operation group, UUO model mice showed significantly lower mRNA and protein expression levels of TJP1 in renal tissue. Transfection with miR-185-5p agomir led to further reduction of the mRNA and protein expression levels of TJP1 in renal tissue (**Figure 4B–D**), whereas the expression levels of α-SMA, fibronectin, COL-I, and COL-III were significantly increased (**Figures 6C, D**). Transfection with miR-185-5p antagomir partially restored the mRNA and protein expression levels of TJP1 in renal tissue (**Figures 6B–D**), thereby reducing the expression levels of α-SMA, fibronectin, COL-I, and COL-III (**Figures 6C, D**). These findings indicate that miR-185-5p overexpression can reduce TJP1 expression and exacerbate renal tubulointerstitial fibrosis *in vivo*.

## 4 Discussion

This study found that urinary miR-92a-3p, miR-425-5p, and miR-185-5p levels were significantly higher in IgAN group than in the healthy control; all three miRNAs were associated with tubular atrophy/interstitial fibrosis in IgAN. Importantly, these three urinary miRNAs could predict the extent of tubular atrophy/interstitial fibrosis in patients with IgAN. Our previous cohort study involving 52 patients with IgAN also showed that urinary miR-205 and miR-21 could distinguish between severe and mild renal tubulointerstitial injury, suggesting that urinary sediment miRNAs could serve as non-invasive biomarkers for IgAN tubular atrophy/interstitial fibrosis severity. Another study showed that the expression level of urinary miR-185-5p was significantly lower in patients with diabetes lacking renal injury (i.e., patients without urinary protein and with normal renal function) than in patients with diabetic nephropathy, membranous nephropathy, or IgAN; the expression level of urinary miR-185-5p was highest in patients with IgAN (28). However, the expression level of urinary miR-185-5p in patients with diabetic nephropathy was not associated with renal tubulointerstitial lesions.

Tubulointerstitial fibrosis is the most reliable histological marker of an adverse outcome (2). Multiple IgAN prognosis models, including IIgANPT, have shown that tubular atrophy/interstitial fibrosis is the most important prognostic pathological indicator (3, 4). The severity of tubulointerstitial damage in IgAN is closely correlated with the rate of renal function decline and long-term renal outcomes (5). Non-invasive clinical biomarkers are needed for prediction of tubular atrophy/interstitial fibrosis in patients with IgAN. Our results showed that urinary miR-92a-3p, miR-425-5p, and miR-185-5p can serve as specific biomarkers for tubular atrophy/interstitial fibrosis in patients with IgAN; combination of these three miRNAs yielded an AUC of 0.742. Patients with a high expression level of urinary miR-185-5p have a poor renal prognosis. The level of urinary miR-185-5p is associated with low osmotic pressure and elevated NAG in urine, suggesting that urinary miR-185-5p is associated with renal tubulointerstitial injury. *In situ* hybridization of renal tissue from patients with IgAN showed that miR-185-5p was mainly localized in renal tubular epithelial cells. A previous study showed that the expression of miR-185-5p in renal tissue is significantly higher among patients with IgAN than among participants in a normal control group (29). Our findings indicate that miR-185-5p is mainly derived from renal tubular epithelial cells; the urinary miR-185-5p level can be used to predict the presence of tubular atrophy/interstitial fibrosis in patients with IgAN, enabling non-invasive prognostic evaluation for patients with IgAN who display renal impairment. We previously conducted a cohort study involving 52 patients with IgAN, which showed that the miR-21 level can be used to predict IgAN T grade with an AUC of 0.74. TGF-β 1 can stimulate miR-21 expression (30, 31), suggesting that miR-21 is located downstream of TGF-β1. The predictive relationship of miR-21 with tubular atrophy/interstitial fibrosis in IgAN may be related to its relationship with TGF-β1. However, the present findings suggest that no known contributing factor can stimulate the expression of miR-185-5p; thus, the increase in miR-185-5p expression may be attributed to non-TGF-β1 pathways. Moreover, miRNA sequencing of renal tissue from UUO model mice showed no significant difference in miR-185-5p expression between obstructed and healthy kidneys (31).

Analyses of GO biological processes and KEGG pathways involving miR-185-5p target genes suggested that the following critical pathways influenced renal fibrosis: cell−cell adhesion, actin cytoskeleton regulation, focal adhesion, and adherens junctions. Protein-protein interaction network construction and dual luciferase assays indicated that TJP1 is a hub gene for miR-185-5p. Mass spectrometry analysis of renal tissue proteins collected from patients with IgAN in our previous study revealed that the expression of TJP1 was significantly lower in the IgAN group than in the normal control group (27). Within renal tissues, TJP1 is mainly expressed in podocytes, proximal tubules, distal convoluted tubules, and collecting ducts (32). Tight junctions are key structures that anchor adjacent tubular epithelial cells, an essential interaction for the maintenance of tubular barrier integrity (32). Abnormal expression of tight junction proteins is associated with acute kidney injury, and restoration of tight junction protein expression through various mechanisms can alleviate the renal dysfunction involved in acute kidney injury (33). Moreover, TJP1 establishes a framework for podocyte connection, while playing a critical role in renal development and the maintenance of renal function (34). TJP1 expression in podocytes and its interactions with slit diaphragm components (e.g., nephrin, NEPH1, and NEPH3) are necessary to maintain glomerular filtration barrier integrity (35). Selective knockout of TJP1 in renal podocytes can cause severe proteinuria in mice, leading to renal impairment and global sclerosis (36). The present study showed that miR-185-5p mimic can reduce expression of the target gene TJP1; increase the levels of α-SMA, fibronectin, COL-I, and COL-III; and promote the transformation of renal tubular epithelial cells to a fibrotic phenotype. Moreover, miR-185-5p inhibitor could reverse the fibrotic phenotype among renal tubular epithelial cells stimulated with TGF-β1. Experiments with the UUO mice model confirmed that tubular atrophy/interstitial fibrosis could be alleviated by inhibiting miR-185-5p expression. Overall, the present study provided novel therapeutic insights for reducing tubular atrophy/interstitial fibrosis in patients with IgAN.

This study had some limitations. First, it utilized a single-center design and only included Chinese patients. Notably, the incidence and clinical indicators of IgAN differ among countries, and IgAN prognoses differ according to ethnicity (4). In future studise, the present findings should be confirmed using IgAN cohorts from multiple centers and various countries. Second, the combination of the three miRNAs predicted IgAN tubular atrophy/interstitial fibrosis with an AUC of 0.742, similar to previous findings regarding urinary miR-205 and miR-21 and have not improved (18). Because tubular atrophy/interstitial fibrosis involves multiple pathways (e.g., inflammation, apoptosis, aging, and oxidative stress), it is difficult to fully represent this complex process using a single pathway and individual biomarkers. Our findings suggest that a model can be established using multiple non-invasive biomarkers, along with various clinical indicators, to improve the sensitivity and specificity of tubular atrophy/interstitial fibrosis prediction.

The expression levels of urinary miR-92a-3p, miR-425-5p, and miR-185-5p were significantly increased in patients with IgAN; all were associated with tubular atrophy/interstitial fibrosis in such patients. miR-185-5p may facilitate the transformation of renal tubular epithelial cells to fibrotic phenotype through its target gene TJP1, thereby affecting IgAN prognosis.

## Data availability statement

The original contributions presented in the study are included in the article/**Supplementary Material**. Further inquiries can be directed to the corresponding author.

## Ethics statement

The studies involving humans were approved by the ethics committee of the Chinese People’s Liberation Army General Hospital (Beijing, China). The studies were conducted in accordance with the local legislation and institutional requirements. The participants provided their written informed consent to participate in this study. The animal study was approved by Experimental Animal Ethics Committee of Taikang Medical Laboratory Services Hebei Co., Ltd. The study was conducted in accordance with the local legislation and institutional requirements.

## Author contributions

ZD: Data curation, Formal analysis, Project administration, Validation, Writing – original draft, Writing – review & editing. RB: Data curation, Methodology, Project administration, Writing – original draft. SL: Investigation, Software, Writing – original draft. XC: Formal analysis, Supervision, Writing – original draft. CZ: Data curation, Methodology, Writing – original draft. QZ: Formal analysis, Methodology, Software, Writing – original draft. JL: Supervision, Validation, Writing – review & editing. XC: Conceptualization, Resources, Writing – review & editing. GC: Conceptualization, Funding acquisition, Investigation, Resources, Supervision, Writing – review & editing.

## Glossary

**Table:**

IgAN	IgA nephropathy
TJP1	tight junction protein 1
RaDaR	United kingdom National Registry of Rare Kidney Diseases
IIgANPT	international IgAN prediction tool
TGF-β1	transforming growth factor-β1
SMAD3	mothers against decapentaplegic homolog 3
eGFR	estimated glomerular filtration rate
PCR	polymerase chain reaction
miRNA	microRNA
UUO	unilateral ureteral obstruction
GAPDH	glyceraldehyde-3-phosphate dehydrogenase
NAG	β-N-acetyl glycosaminidase
CKD-EPI	Chronic Kidney Disease Epidemiology Collaboration
UTR	untranslated region
α-SMA	α-smooth muscle actin
COL-I	collagen I
COL-III	collagen III
ROC	Receiver operating characteristic
AUC	areas under the ROC curve
NC	normal control group
DC	disease control group
GO	gene ontology
KEGG	kyoto encyclopedia of genes and genomes
TJP1-MUT	TJP1-mutant type
TJP1-WT	TJP1-wild type;
