# Supplementary material for: Urinary miR-185-5p is a biomarker of renal tubulointerstitial fibrosis in IgA nephropathy
Source: Front Immunol. 2024 Feb 15;15:1326026. doi: 10.3389/fimmu.2024.1326026 (PMC10902439; doi:10.3389/fimmu.2024.1326026)

Table S1. Urinary miR-92a-3p, miR-425-5p, and miR-185-5p expression levels in two IgAN profiles.

|  | LEE I-II vs NC | | LEE III vs NC | | LEE IV-V vs NC | |
| --- | --- | --- | --- | --- | --- | --- |
|  | Duan et al. 2016 | Wang et al. 2015 | Duan et al. 2016 | Wang et al. 2015 | Duan et al. 2016 | Wang et al. 2015 |
| miR-92a-3p (Fold change) | 216.72 | 5.87 | 185.41 | 4.47 | 83.02 | 4.18 |
| P value | 0.029 | 0.027 | 0.022 | 0.104 | 0.034 | 0.198 |
| miR-425-5p (Fold change) | 249.75 | 10.14 | 147.87 | 10.36 | 49.74 | 9.64 |
| P value | 0.004 | 0.006 | 0.002 | 0.042 | 0.011 | 0.145 |
| miR-185-5p (Fold change) | 888.18 | 6.41 | 478.87 | 7.56 | 165.55 | 5.79 |
| P value | <0.001 | 0.06 | 0.008 | 0.141 | 0.013 | 0.199 |

Fold change: IgAN/Normal control group; LEE: Lee’s grade of IgAN; NC: Normal control group.

Table S2. Clinical and demographic characteristics of patients in IgAN and control groups.

|  | IgAN  (n=188) | NC  (n=33) | P value |
| --- | --- | --- | --- |
| Male[n(%)] | 95(50.53) | 17(51.52%) | 0.917 |
| Age(years) | 33.50(27.00-41.00) | 37.00(32.00-41.00) | 0.194 |
| MAP(mmHg) | 96.67(89.67-103.33) |  |  |
| Urine Osmolality (mOsm/kg·H_2_O) | 611.94±206.05 |  |  |
| Alb(g/L) | 38.70(35.70-41.40) |  |  |
| Scr(μmol/L) | 93.70(73.90-132.50) |  |  |
| UA(μmol/L) | 367.88±107.33 |  |  |
| Cys C(mg/L) | 1.05(0.84-1.53) |  |  |
| eGFR(ml/min/1.73m^2^) | 87.18(59.35-109.19) |  |  |
| Hematuria(%) | 82.45 |  |  |
| Proteinuria(g/24h) | 1.27(0.63-2.34) |  |  |

NC: normal control; MAP: mean arterial pressure; Scr: serum creatinine; UA: uric acid; Cys C: cystatin C; eGFR: estimated glomerular filtration rate.

Figure S1. Co-prediction of target genes by three databases.


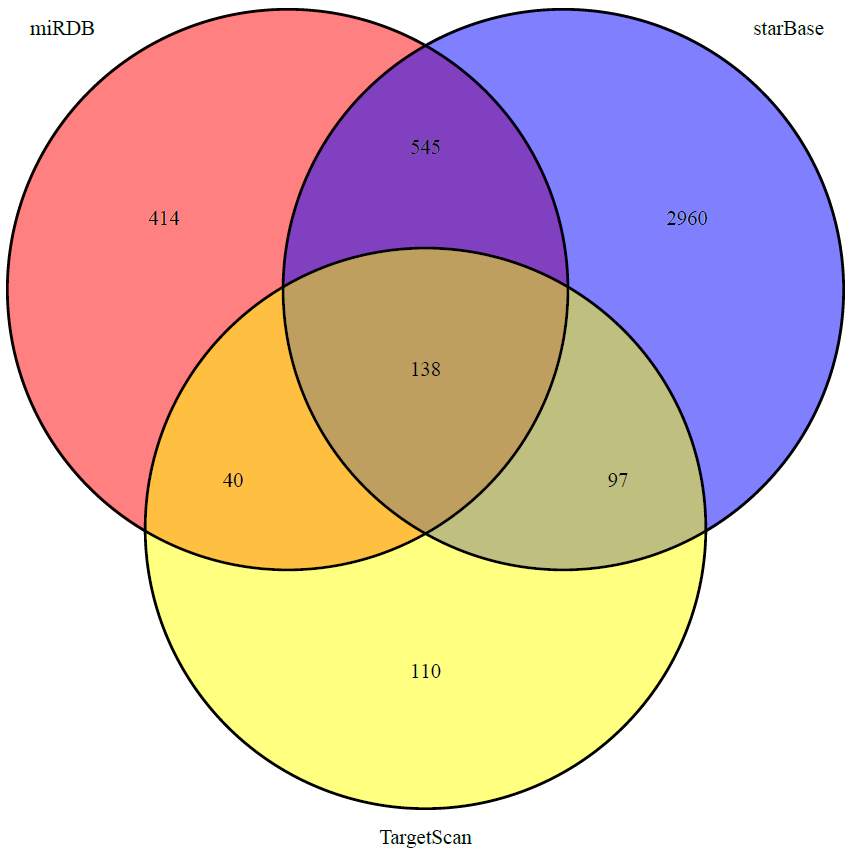


Figure S2. GO analysis of processes associated with miR-185-5p.


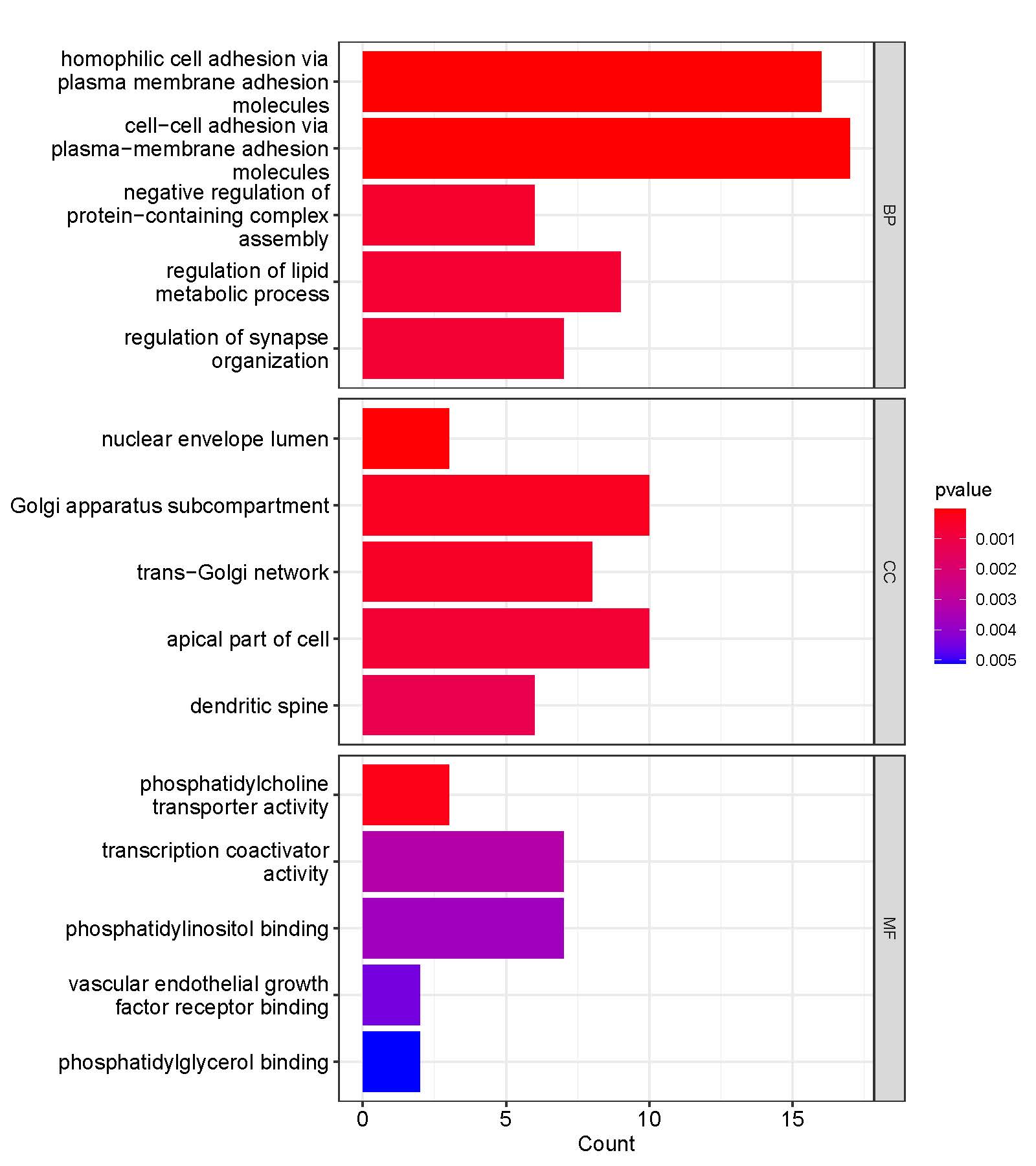


Figure S3. KEGG analysis of pathways associated with miR-185-5p.


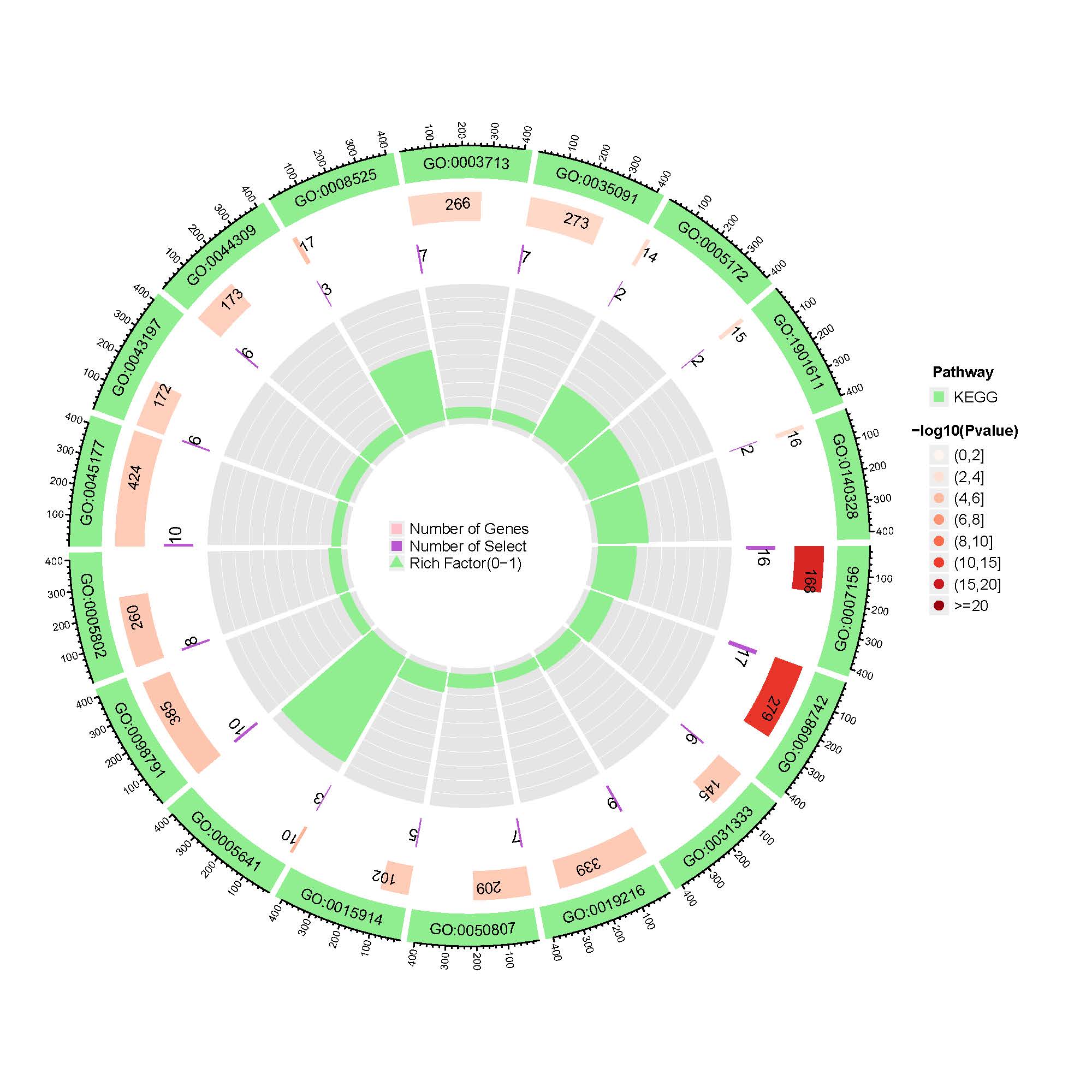


Figure S4. Luciferase reporter assays of the interaction between miR-185 and TJP1.


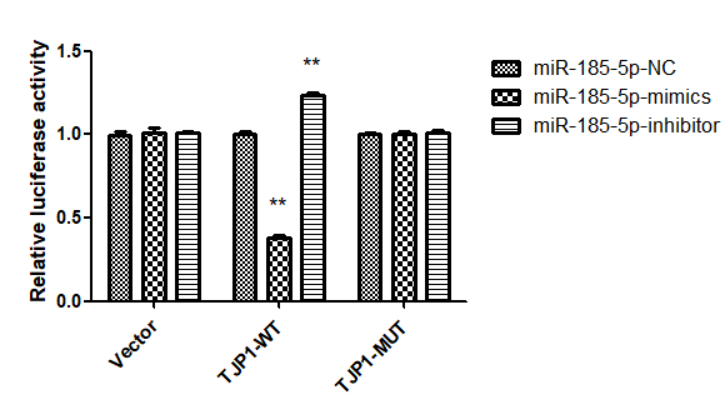


TJP1-MUT: Mutant PmirGLO-TJP1; TJP1-WT: Wild-type PmirGLO-TJP1; ** P<0.001

Figure S5. Fluorescence in situ hybridization of miR-185.


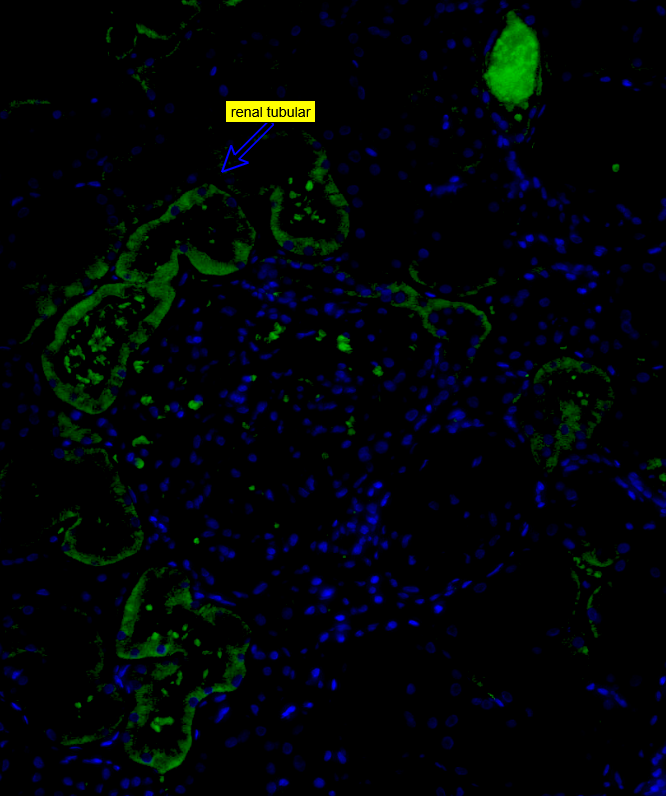

Supplement: Supplementary file 1 [file DataSheet_1.docx]
